# Plasticity and Constraints of tRNA Aminoacylation Define Directed Evolution of Aminoacyl-tRNA Synthetases

**DOI:** 10.3390/ijms20092294

**Published:** 2019-05-09

**Authors:** Ana Crnković, Oscar Vargas-Rodriguez, Dieter Söll

**Affiliations:** 1 Department of Molecular Biophysics and Biochemistry, Yale University, New Haven, CT 06520, USA; oscar.vargas@yale.edu (O.V.-R.); dieter.soll@yale.edu (D.S.); 2 Department of Chemistry, Yale University, New Haven, CT 06520, USA

**Keywords:** aminoacyl-tRNA synthetases, noncanonical amino acids, directed evolution, genetic code expansion, synthetic biology

## Abstract

Genetic incorporation of noncanonical amino acids (ncAAs) has become a powerful tool to enhance existing functions or introduce new ones into proteins through expanded chemistry. This technology relies on the process of nonsense suppression, which is made possible by directing aminoacyl-tRNA synthetases (aaRSs) to attach an ncAA onto a cognate suppressor tRNA. However, different mechanisms govern aaRS specificity toward its natural amino acid (AA) substrate and hinder the engineering of aaRSs for applications beyond the incorporation of a single l-α-AA. Directed evolution of aaRSs therefore faces two interlinked challenges: the removal of the affinity for cognate AA and improvement of ncAA acylation. Here we review aspects of AA recognition that directly influence the feasibility and success of aaRS engineering toward d- and β-AAs incorporation into proteins in vivo. Emerging directed evolution methods are described and evaluated on the basis of aaRS active site plasticity and its inherent constraints.

## 1. Introduction

Synthesis of proteins with exotic building blocks, noncanonical amino acids (ncAAs), can be achieved in vivo and in vitro. However, the repertoire of ncAAs that can be introduced into peptides and proteins in vitro is larger than in vivo, due to the fact that the aminoacyl (AA)-tRNA substrates can be generated by Flexizyme, an artificial ribozyme with no ncAA specificity [1]. In vitro studies have also revealed a fairly accommodating nature of the ribosome, which can allow introduction of highly diverged ncAAs, with little [2,3] or no modification [4,5]. In vivo, however, ncAA-tRNAs have to be synthesized by aminoacyl-tRNA synthetases (aaRSs) which have been naturally optimized during evolution to stringently recognize only one, canonical amino acid [6]. On the other hand, the majority of ncAAs have not been present in the course of natural evolution and tRNA synthetases have not yet evolved to reject them; this fact is exemplified by the apparent flexibility of wild-type synthetases which can, in vitro, aminoacylate even *N*-methylated and β-amino acids, as well as α, α-disubstituted amino acids [7]. Supplementation based methods exploit this flexibility of natural aaRSs [8] but 100% incorporation of ncAA may not be feasible using this technology. An ncAA is aminoacylated by a wild-type aaRS and introduced in a residue-specific manner; the matching natural amino acid concentration is kept low by the use of a cAA-auxotrophic strain, while the ncAA is added to the growth medium. However, the target protein must be further mutated in order to achieve site-specific incorporation (e.g., for selenocysteine (Sec) incorporation by cysteinyl-tRNA synthetase (CysRS) and in response to cysteine (Cys) codons, non-target Cys residues need to be replaced with serine (Ser) [9]; similarly, for incorporation of fluorinated tryptophan (Trp) analogs, non-target Trp residues need to be mutated to tyrosine (Tyr) [10].

Yet, even with the use of an auxotrophic strain, some natural amino acid is still present in the cellular pool and the aaRS will preferentially activate it. This effectively lowers the extent of ncAA insertion. Thus, to generate a target protein decorated with an ncAA at a chosen position, and without additional mutations, one needs to employ the nonsense-suppression method [8]. To that end, an orthogonal aaRS(o-aaRS)·tRNA_CUA_ pair needs to be introduced to the cell, and the targeted position in the protein of choice mutated to the amber (TAG) stop codon. An aaRS is orthogonal if it does not react with any of the tRNAs in the host; conversely, an orthogonal tRNA will not be a substrate for the host’s aaRSs. The determinants governing aaRS·tRNA_CUA_ orthogonality have been reviewed elsewhere in detail [11] and will not be discussed here. With intention to present the current state and advances in directed evolution of aaRSs we will focus on aspects of AA/ncAA recognition and ncAA availability in cellulo.

## 2. Flexibility of Wild-Type Synthetases

### 2.1. Implications of Substrate Recognition and Catalysis for Engineering of aaRSs

**Figure 1:**
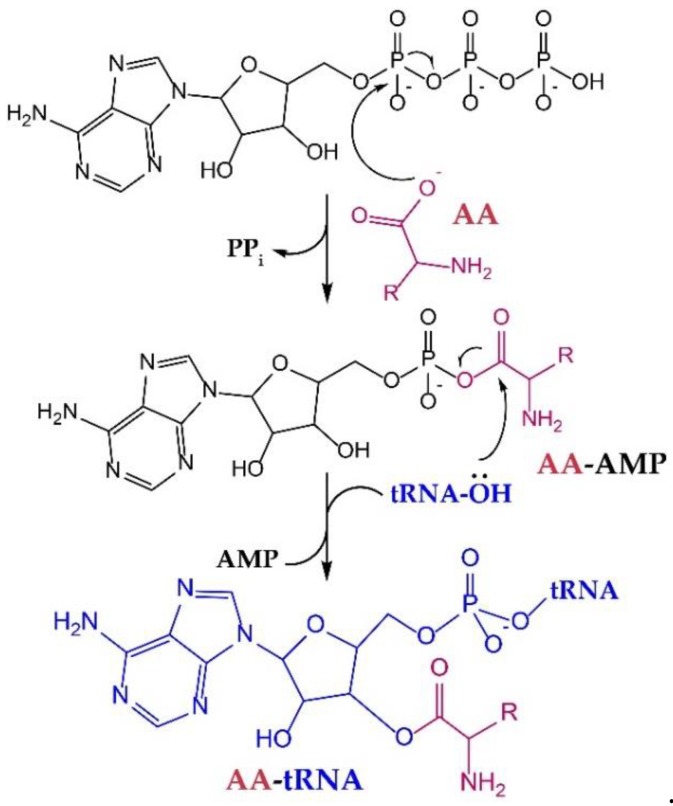
Aminoacyl-tRNA synthetases (aaRSs) catalyze aminoacyl (AA)-tRNA formation in two steps. First, the aminoacyl adenylate (AA-AMP) is formed and the pyrophosphate (PP_i_) is released. Next, the oxygen atom from the ribose hydroxyl group of the tRNA attacks the carbonyl carbon of the AA-AMP, forming AA-tRNA with the release of AMP.

All aaRSs catalyze aminoacylation in a two-step reaction, wherein the AA is first “activated” by condensation with ATP and then transferred to the 3′-terminal adenosine of tRNAs in the second step (Figure 1). Mechanistically, aaRSs use binding energy between active-site residues and the substrates to gain specificity and enhance the rate of catalysis [12]. Moreover, although only the last three nucleotides of the tRNA (the conserved 3′-CCA terminus) enter the active site, recognition of the tRNA anticodon, which occurs ~40 Å away from the active site, is essential for proper organization of the active site. Thus, mutations in the anticodon of a tRNA [13] to create a suppressor tRNA do not only reduce its affinity with the aaRS [14], but can also impact AA binding and aminoacylation efficiency [15]. In some cases, aaRS·tRNA complex formation is required for AA or ATP binding [16]. Interestingly, some of these aaRSs retain the capacity to aminoacylate tRNA anticodon variants in vivo with no additional mutations to the anticodon-binding domain [17,18]. Therefore, the effect of tRNA mutations on the efficiency of the aminoacylation reaction may vary in severity between different species [18], or can simply be compensated by aaRS overexpression and, possibly, by other unknown mechanisms [17].

It is considered that aaRSs stabilize the transition state of both steps of aminoacylation [19], which, for the purposes of aaRS engineering, may confound the visual inspection of the active site and the roles of individual residues within it. Thus, although structures of aaRS·AA and aaRS·ncAA complexes reveal significant differences in the hydrogen bonding patterns and steric interaction between wild-type aaRS·AA and aaRS·ncAA complexes [20], separating the specific role of active-site residues in initial substrate binding and stabilization of the transition state remains challenging. Sophisticated analysis and redesign of the active site may therefore require >15 residues to be mutagenized around the active site alone, in order to create a superior, ncAA-specific aaRS [21]. Concomitantly, more structural data may be needed to aid the design and discovery of improved, second-generation aaRS variants.

### 2.2. The Inherent AA Polyspecificity of aaRSs

**Figure 2:**
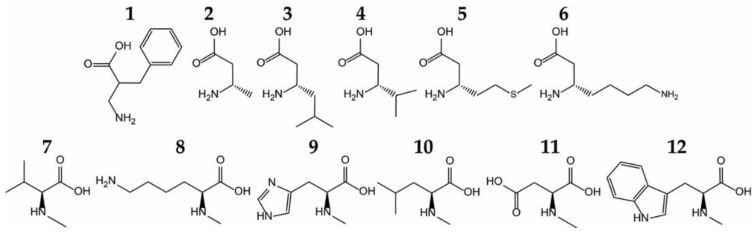
Examples of noncanonical amino acids (ncAAs) acylated by wild-type aaRSs in vitro. Phenylalanyl-tRNA synthetase (PheRS) acylates a β_2_-amino acid, 2-aminomethyl-3-phenyl-propionic acid (**1**). Five β_3_-amino acids (Ala (**2**), Leu (**3**), Val (**4**), Met (**5**), and Lys (**6**), are substrates for their cognate aaRSs. *N*–methylated amino acids that are also substrates for wild-type aaRSs include *N*-methyl-valine (**7**), *N*-methyl-lysine (**8**), *N*-methyl-histidine (**9**), *N*-methyl-leucine (**10**), *N*-methyl-aspartate (**11**), and *N*-methyl-tryptophan (**12**) [7].

AaRSs recognize the unique side chain of their cognate AA with high specificity, both sterically and through hydrogen bonds and salt bridges. In addition, at least half of the aaRSs also possess a conserved acidic (Asp/Glu) residue that binds the α-amino group. However, this specificity was developed with regard to other natural AAs. Actually, when tested against different unnatural ncAAs, aaRSs show remarkable flexibility in ncAA recognition, and can aminoacylate these ncAAs in vitro [7] and in vivo [22] (Figure 2). The most surprising ncAA substrates include some bulky ncAAs, *N*-methylated and β-amino acids, as well as α, α-disubstituted amino acids [7,22,23].

### 2.3. Recognition of Fluorinated AA Analogs

Fluorinated AAs have been widely used for protein labeling purposes in *Escherichia coli* auxotrophic strains, through missense suppression [23]. Interestingly, these isosteric ncAAs may be rejected. For instance 3′-trifluorinated isoleucine (Ile) cannot be aminoacylated by *E. coli* isoleucyl-tRNA synthetase (IleRS) [24]. Similarly, hexafluoroleucine incorporation in vivo requires leucyl-tRNA synthetase (LeuRS) overexpression to overcome impaired aminoacylation [25]. On the other hand, fluorinated histidine (His) is normally aminoacylated by histidyl-tRNA synthetase (HisRS) [26]. Fluorinated Trp analogs are readily accepted by bacterial tryptophanyl-tRNA synthetases (TrpRSs) [27,28], as well as Tyr derivatives [22,29]. Both 4-fluorinated and 4,4-difluorinated glutamate (Glu) can be aminoacylated in vitro and introduced into proteins in vivo [7,30].

### 2.4. Improving ncAA-tRNA Synthesis Using AA-tRNA Deacylases

Almost half of the aaRSs mis-aminoacylate other natural AAs. They also possess a mechanism to clear (edit) erroneously formed aminoacyl adenylate or mis-aminoacylated tRNA [16]. The latter reaction is catalyzed by a separate “editing” domain. The hydrolytic site of the editing domain can catalyze deacylation of noncognate natural AAs, as well as some ncAAs. For instance, while trifluoroethylglycine can be acylated by *E. coli* IleRS without any modification to the synthetic active site; it does, however, require a mutation in the editing domain that prevents hydrolysis of the trifluoroethylglycyl-tRNA [31]. Similarly, experiments with an editing-deficient variant of valyl-tRNA synthetase revealed that the enzyme is capable of acylating >10 different ncAAs including α,α-disubstituted and cyclic β-amino acids [32]. Therefore, accurately identifying the activities of these editing domains in aaRS reaction with an ncAA may be critical for the ncAA incorporation into proteins.

Editing domains have been exploited to increase the purity of ncAA-tRNA synthesis in vivo and in vitro. For example, the editing domain of phenylalanyl-tRNA synthetase (PheRS), which naturally deacylates Tyr-tRNA^Phe^ [33], has been fused to an *E. coli* TyrRS variant designed to acylate 3-iodo-l-tyrosine. The appended domain allows the mutant enzyme to clear the residual affinity for Tyr by specific hydrolysis of Tyr-tRNA^Tyr^. Thus, without additional mutations to the synthetic site, the accurate synthesis of 3-iodo-Tyr-tRNA was increased [34]. Interestingly, this editing domain of archaeal PheRS also shows stereospecificity, and hydrolyzes l-Tyr-tRNA, but not d-Tyr-tRNA. A TyrRS-PheRS chimera has been shown to possess increased enantioselectivity toward d-Tyr-tRNA^Tyr^ [35]. A similar approach was used to design a human mitochondrial PheRS variant that is “resistant” to misacylation of the reactive oxygen species (ROS) derived from Phe (such as Tyr and *m*-Tyr). The mitochondrial version inherently lacks the editing domain but discriminates between Tyr and Phe with a factor of 10^3^. Human cytosolic PheRS possesses an editing domain, but it cannot clear *m*-tyrosyl-tRNA^Phe^. By fusing the editing domain of *E. coli* PheRS a variant in which Tyr-tRNA^Phe^ and *m*-Tyr-tRNA^Phe^ can be enzymatically deacylated was created [36].

### 2.5. Evolvability of 20 aaRSs

Although more than 150 ncAAs have been adapted for in vivo incorporation to date [23], the vast majority are activated by only two orthogonal aaRSs: archaeal TyrRS and pyrrolysyl-tRNA synthetase (PylRS). Both synthetases encompass some of the critical features that a synthetic-biology ready aaRS needs: good orthogonality [37,38] and a flexible active site [39]. Another useful feature of PylRS is that it does not recognize the tRNA anticodon [37,40]. Both enzymes possess a spacious active site that can be mutagenized with relative ease in order to facilitate incorporation of bulky ncAAs. PylRS even has a flexible mode of α-amino group recognition, thereby allowing activation of β-substituted amino acids [41]. TyrRS active site has been proven extremely flexible, possibly due to similarities with TrpRS [42].

Are aaRSs in general amenable to laboratory design and directed evolution? If we consider that “A good parent protein for directed evolution…exhibits enough of the desired function that small improvements (expected from a single mutation) can be reliably discerned in a high-throughput screen” [43], then the answer is yes, especially since many wild-type aaRSs can acylate divergent amino acid substrates in vitro. Also, the majority of evolved aaRS variants contain only a few mutations that improve their activity for a desired ncAA. The problem may lie more in the nature of assays/reporters employed to screen for superior variants. So far, aaRSs have been evolved using reporters/assays that tie the success of ncAA incorporation into proteins to the success of ncAA aminoacylation. Due to such setup we may have failed to identify aaRS variants with improved affinity toward such exotic AAs that are rejected by other components of the translation apparatus (e.g., elongation factor EF-Tu/EF1a or the ribosome), rather than the aaRS itself. Furthermore, because directed evolution of aaRSs has been almost ubiquitously employed in vivo, we may have overlooked prospective aaRS candidates with starting ncAA affinities that cannot be achieved intracellularly, or those that cannot be tested in vivo, due to their lack of orthogonality.

### 2.6. Availability of ncAA In Vivo May Dictate ncAA Incorporation

Affinity of aaRSs for their cognate amino acid is typically in a micromolar range, but can vary between different aaRSs up to 100-fold. In organisms where the absolute concentration of an amino acid has been measured, and the corresponding aaRS biochemically characterized, we can observe that the amounts of free AA are several fold higher than the *K*_M_ (AA) for this synthetase. Affinity toward cognate AA is frequently investigated as a part of general kinetic studies and Michaelis constants for AA substrates are readily available for all 21 aaRSs. Absolute concentrations of amino acids in a glucose-fed, exponentially growing *E. coli* show that aaRSs affinity matches the intracellular concentration of its cognate AA and that individual AA concentrations exceed the *K*_M_ (AA) of a given aaRS [44]. For instance, *E. coli* TyrRS catalyzes Tyr-AMP formation with a *K*_M_ (Tyr) of 7.13 ± 0.81 µM [45]. On the other hand, *E. coli* AlaRS catalyzes Ala-AMP formation with almost 100 times lower affinity (*K*_M_ (Ala) 300 ± 120 μM). Although these values show a wide range of affinities toward cognate AA, they are consistent with intracellular AA concentration, where Ala is 100 times more abundant than Tyr (2.6 mM vs. 29 μM, respectively [44]).

Similarly, for the purpose of directed evolution in vivo, the intracellular concentration of an ncAA needs to match the aaRS initial affinity. The initial prerequisites are therefore intertwined: the intracellular supply of an ncAA must compensate for the aaRS affinity towards it. Wild-type *E. coli* TyrRS has a *K*_M_ (azatyrosine) of 17.7 ± 2.9 μM, which is only around five times lower in affinity compared to cognate Tyr; by adding 50 µM azatyrosine to the growth medium, a significant fraction of the cellular proteins will be labeled with this ncAA [46]. On the other hand, the archaeal *O*-phosphoseryl-tRNA synthetase (SepRS) accepts its cognate AA phosphoserine (Sep) with much lower affinity (*K*_M_ (Sep) of 270 ± 40 μM for Sep-AMP formation) [47]. At the same time, intracellular levels of Sep in wild-type *E. coli* grown in rich medium are below the detection limit; miniscule amounts of intracellular Sep are observed only when 5 mM Sep is added to the culture [48].

Sufficient intracellular levels of ncAA can be achieved by supplementation of such ncAAs to the growth media. In that case, the ncAA is imported via canonical transport systems [49]. In cases where the physicochemical properties of the ncAA prevent its recognition by the carrier proteins or its diffusion across the membrane, the ncAA may be supplied covalently linked to a natural AA and introduced to the cell through portage transport [50]. Binding proteins of *E. coli* oligo- and dipeptide permease systems show broad substrate specificity and are able to accept a wide range of peptide substrates [51]. This promiscuity of peptide import has been exploited to transport *O*-phosphotyrosine and its derivatives into *E. coli*, thereby allowing identification of an aaRS capable of acylating *O*-phosphotyrosine [52]. Interestingly, the binding protein of the OppABCDF permease system has been shown to recognize the backbone of the oligopeptide, therefore limiting the spectrum of ncAA that could be introduced to the cytosol in this manner [53]. Recently, an interesting approach to circumvent this issue was reported [54]. This strategy combines the promiscuity of peptide import with the activity of a cytosolic enzyme, γ-glutamyl transferase. The ncAA is attached to the γ-carboxyl group of glutamate residue of an oligopeptide. Upon import, the ncAA is liberated through the action of cytosolic γ-glutamyl transferase. This approach may be useful for the import of ncAAs with alternative backbones [54].

Alternatively, some ncAAs can be produced intracellularly by manipulation of the host genome. For instance, increased intracellular levels of Sep can be achieved by the deletion of phosphoserine phosphatase gene, both in *E. coli* [48,55] and HEK293 cells [56]. A more complex strategy needs to be employed for ncAAs that are not normally part of the cellular metabolome. This may require introduction of complete biosynthetic operons (as with pyrrolysine (Pyl) [57]) or unique genes from another organism. For example, to generate sufficient amounts of *O*-phosphothreonine (pThr) in *E. coli* it was necessary to introduce the threonine kinase gene from *Salmonella enterica*, which catalyzes pThr formation using threonine (Thr) and ATP as substrates [58]. High expression levels of this enzyme in *E. coli* is necessary to achieve intracellular concentrations of up to 1.7 mM [59]. Although SepRS shows lower affinity for pThr than 1.7 mM (*K*_M_ (pThr) in aminoacylation 8.4 ± 4.0 mM) [47]; these intracellular levels were sufficient to select for a variant capable of introducing pThr into proteins in *E. coli* [59].

How well can we predict intracellular concentration of a desired ncAA? Initial tests to explore the substrate range of an aaRS include selection/screening outcomes in the presence and absence of an ncAA. However, improved synthesis of a reporter in the presence of the ncAA may not necessarily signal ncAA acylation unless the reporter is sensitive/specific to ncAA insertion. Some ncAAs may be metabolically active leading to a decrease of their intracellular level and/or decreased survival due to the toxicity of their byproducts [60]. The function of ncAAs as allosteric inhibitors in cellular biosynthetic pathways for the similar natural AAs may be conceivable, although it is fairly unexplored [61]. The choice of growth media may also influence correct identification of ncAA substrates. For instance, phosphorylated ncAAs like Sep and pThr enter *E. coli* via a complex phosphonate transporter whose expression is induced by phosphate limitation [48,62]. In cases where a satisfactory intracellular concentration of ncAA cannot be achieved, ancestral functions may become prominent and lead to aaRS variants capable of noncognate, natural AA acylation [63].

## 3. Directed Evolution of Orthogonal tRNA Synthetases

Directed evolution of aaRSs is typically conducted in vivo and for that reason the principal requirement is the aaRS and tRNA orthogonality, i.e., the absence of cross-talk with endogenous aaRSs and tRNAs. Generally, the orthogonal tRNA is set to decode the amber (UAG) stop codon by mutating its anticodon to CUA. Successful acylation of the orthogonal tRNA_CUA_ is monitored by UAG read-through, where the reporter gene contains an in-frame UAG codon.

As with other enzymes, the success of directed evolution rests on the quality/diversity of aaRS libraries and an unambiguous phenotype that allows rapid and accurate detection of proficient aaRS variants. Commonly used reporters for identification of aaRS candidates, chloramphenicol resistance [64] and GFP fluorescence [65], have been adapted for the directed evolution of aaRSs.

### 3.1. Sequence Randomization Methods

A good, rationally designed aaRS library requires accurate identification of active site residues that participate in substrate binding and/or catalysis (see above). These residues are the most frequent targets for saturation mutagenesis where every possible AA substitution is tested simultaneously. Advances in primer design, most notably in removing codon redundancy that exists with NNK randomization, facilitate screening and allow a more balanced distribution of individual amino acid replacements in the library [66]. However, even with the utilization of non-redundant primers it is not feasible to target more than seven residues (~1.3 × 10^9^ library members) with conventional saturation-mutagenesis approaches. Advanced computational design using Rosetta3 [67] enabled systematic investigation of variants in which 17 residues were targeted but not all substitutions were tested [15]; this approach led to the discovery of TyrRS variant with high specificity for photocaged 3,4-dihydroxyphenyl-alanine (DOPA).

Significant advances have been made in creating aaRS libraries in situ [68,69,70]. These approaches circumvent the challenge of creating plasmid-borne libraries: the demand for high transformation efficiency required for introducing large populations of mutant clones. Multiplex automated genome engineering, MAGE [71], uses a previously established technique that exploits the fact that externally introduced λ-phage protein (protein β) binds and anneals single stranded DNA oligonucleotides to a complementary DNA strand in the vicinity of a DNA replication fork [72]. By transforming oligonucleotides in which the target residue has been randomized, a particular part of the bacterial chromosome can be mutated in a focused fashion. This technique was employed to evolve a genome-borne TyrRS variant able to facilitate introduction of an ncAA in up to 30 individual positions of a reporter protein [68]. One potential disadvantage of the technique are off-target incorporations of mutagenic oligonucleotides. Because several cycles of mutagenesis are needed to create a fully randomized library [68], the resulting strain may have decreased reproductive strength. Nonetheless, the strains are sufficiently healthy to survive the screening process [68,71], crucial to identification of the aaRS variant. One important advantage of the method is that genomic placement of the aaRS variant unmasks subtle differences between gain-of-function phenotypes which are often present with plasmid-borne libraries.

As with any focused-mutagenesis method, the quality of the emerging variants is only as good as the initial design of the library. In other words, if a critical residue is not targeted for mutagenesis, superior variants will not be present in the library. Residues that are essential for stabilizing the transition state, or that participate in long-range signaling between the anticodon-binding domain and the active site may not be apparent from the crystal structure. In addition, rational design does not include improvements in aaRS expression and folding in the host.

Although conventional methods to create randomly mutated aaRS sequences via error-prone PCR have been employed with success [73,74], controlled random mutagenesis in vivo allows a less labor-intensive library creation and direct coupling between mutational and selection cycles [75]. Convenient constructs for achieving tunable mutation rates in vivo are the MP-plasmids that contain various mutator genes placed under the control of an arabinose promoter [75]. These constructs have been employed in combination with phage-assisted continuous evolution technique (PACE [76]) to rapidly identify PylRS variants with improved kinetic parameters.

### 3.2. Selection Methods

Selection methods employed for aaRS evolution traditionally link ncAA insertion into a protein with cell survival. Typically, the chloramphenicol acetyltransferase (CAT) gene is used and a stop codon is placed at position 112; aaRS variants that facilitate stop-codon read-through are identified by growth on chloramphenicol, and in the presence of the ncAA. Because any AA is tolerated at position 112, this positive selection step must inevitably be combined with a negative selection. Toxic gene products such as barnase [77], CcdB toxin [78] or TolC [68] are used to eliminate aaRS variants that facilitate read-through using a natural AA (i.e., false positives). A recent negative selection protocol was developed where a toxic variant of *E. coli* PheRS is expressed by nonspecific stop-codon read-through; this variant acylates tRNA^Phe^ with *p*-Cl-phenylalanine and the incorporation of this amino acid mimic leads to proteome-wide incorporation and ultimately cell death [79]. One significant advantage of this mechanism is the ability to fine-tune the stringency of the negative selection step through changing the concentration of *p*-Cl-phenylalanine in the medium.

Bacteriophage protein pIII needed for phage infectivity was used as a reporter to evolve PylRS using PACE, and the related, non-continuous version of the method [40,69]. Gene III variants with up to three TAG codons were used to link ncAA acylating activity of PylRS with successful phage propagation. Importantly, gene III, normally present in the phage genome, is replaced with the aaRS gene; gene III (with one, two or three TAG codons) is supplied on a plasmid. Alternatively, a less stringent selection can be used where gene III expression is driven from the T7-promoter and the corresponding T7 RNA polymerase is added to the system containing a minimum of 2 TAG in-frame codons. For the purposes of improving the ncAA specificity of a polyspecific *Methanocaldococcus jannaschii* TyrRS variant initially designed to acylate *p*-nitro-l-phenylalanine (*p*-NF), a dominant–negative variant of pIII (pIII-neg) was used to switch its affinity to *p*-iodo-l-phenylalanine and eliminate existing affinity to *p*-NF [69]. The principle of pIII-neg action is to reduce the infectivity of the developing phage, resulting in reduced propagation of pIII-neg synthesizing phages and subsequent removal of the misacylating clone from the lagoon.

### 3.3. Screening Methods

Colorimetric [80], fluorescent [17,68,73,81] and luminescent [69,82] reporters have been adapted for medium/high-throughput screens of ncAA-acylating aaRS libraries. In all cases the reporter gene is translated with in-frame UAG codons and the cells with ncAA-acylating aaRS variants are collected based on the observable color, fluorescence or luminescence. High-throughput screening by fluorescence-activated cell sorting (FACS) allows cross-examination of large populations of cells in a reasonable amount of time [83]. Through alternating rounds of sorting (where highly fluorescent cells are collected when growing in the ncAA-supplemented media, and weakly fluorescent ones are collected when ncAA is absent) aaRS library members with the best activity and/or specificity can be identified [65]. To decrease the risk of collecting of false-positives and improve plasmid maintenance GFP-mediated screening benefits from using one-plasmid systems (GFP, tRNA_CUA_ and aaRS variant all encoded from the same vector) [73,84]. Similarly, genomic integration of the aaRS·tRNA_CUA_ pair leads to a decrease in GFP-reporter yields but unmasks differences in GFP expression that are arising from fluctuating expression levels of aaRS variant [68].

### 3.4. *ncAA*-Specific Reporters

Most engineered aaRSs are polyspecific [85,86,87]. This means that the variant retains the capacity to acylate the original, natural AA [88] or that it can discriminate among similar ncAAs [68,74,85,89]. While negative-selection markers can be optimized against particular natural amino acids [90], ncAA-dependent positive-selection reporters remain largely unexplored. In addition to facilitating the discovery of aaRS variants with increased specificity, ncAA-dependent screens and selection assays allow evolution of aaRS·tRNA pairs with partial orthogonality by eliminating the necessity of the negative selection step.

Selenocysteine (Sec)-dependent reporters: In contrast to other reporters of ncAA insertion, screen/selection methods to improve EF-Tu-dependent Sec insertion [91] have always been specific for this ncAA. This is due to the fact that Sec is synthesized in a two-step manner, where tRNA^Sec^ variant is first misacylated with serine (by seryl-tRNA synthetase, SerRS) and then converted to Sec-tRNA^Sec^ (by selenocysteine synthase SelA) [92]. Because both Ser-tRNA_CUA_ and Sec-tRNA_CUA_ can be accepted by EF-Tu and the ribosome, the reporter protein must be sensitive to Ser vs. Sec insertion. A number of Cys-dependent enzymes have been repurposed for Sec-specific selections due to similarities in chemical properties of Cys and Sec [93,94,95]. However, these reporters are yet to be employed to enhance SerRS activity with tRNA^Sec^ [96].

**Figure 3:**
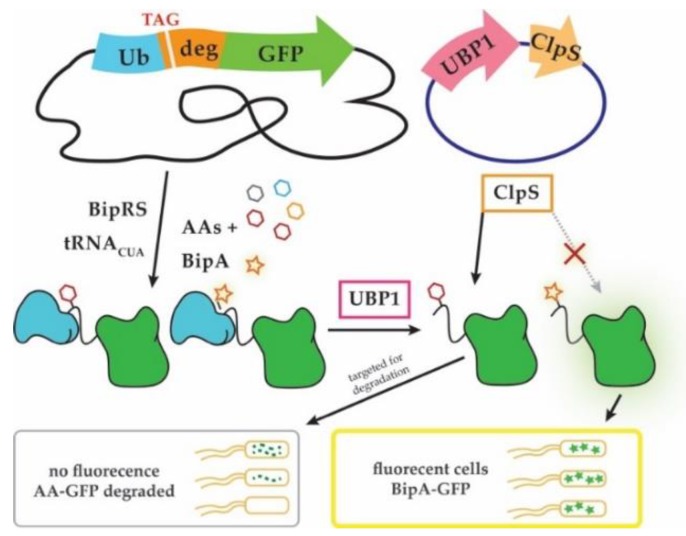
“Posttranslational proofreading” allows monitoring site-specific ncAA incorporation [89]. A ubiquitin-GFP fusion reporter is encoded from the genome, while the necessary enzymes UBP1 and ClpS are overexpressed from a plasmid. The fusion reporter is synthesized via nonsense suppression where different BipRS variants acylate either biphenylalanine (BipA) (star) or a natural AA (hexagons). Ubiquitin-GFP fusions are substrates for UBP1, which removes ubiquitin (Ub) and exposes N-terminally incorporated BipA or natural AA. Truncated fusion proteins that contain destabilizing tyrosine (Tyr), Phe or Leu residues in place of the amber (TAG) stop codon are directed for degradation by ClpS. In contrast, GFP fusions containing BipA are stable and not degraded (see text for details).

Unnatural ncAA-dependent reporters: A recently reported method that allows selective discovery of ncAA-acylating TyrRS variants showed that the activity of protein degradation machinery can be harnessed to specifically identify ncAA insertion in reporter proteins (Figure 3, [89]). The method exploits the N-end rule of protein degradation, which defines the half-life of a protein by the identity of its N-terminal amino acid [97]. The method was used to improve the specificity of the TyrRS variant (BipRS) that acylates biphenylalanine (BipA), and to lesser extent Tyr and several Tyr analogs. The reporter fusion protein contains a cleavable ubiquitin domain on the N-terminus, a UAG codon and a conditionally strong N-degron in the middle, followed by a GFP reporter on the C-terminus. Fluorescence is initially observed nonspecifically with UAG-read-through. However, upon expression of a yeast ubiquitin cleavase protein (UBP1) the fusion protein is cleaved exposing the AA inserted in response to UAG. Because the AA/ncAA now occupies the first position on the N-terminus, it can be recognized by the adaptor protein ClpS, which preferentially binds destabilizing Tyr residues and directs them for degradation. Thus, cells containing aaRS variants that enable BipA insertion will retain fluorescence in UBP1 presence, while those that preferentially facilitate Tyr incorporation will not. Importantly, the most destabilizing residues Tyr, Phe and Leu are also the ones most frequently misincorporated by engineered TyrRS-, PylRS- and LeuRS variants. Furthermore, wild-type ClpS and its variants were shown to have different affinities for chemically similar ncAAs which could be useful to rapidly engineer aaRS variants with unique ncAA specificity [74,89].

### 3.5. Adaptation of Next-Generation Sequencing (NGS) to Sidestep a Negative Selection

**Figure 4:**
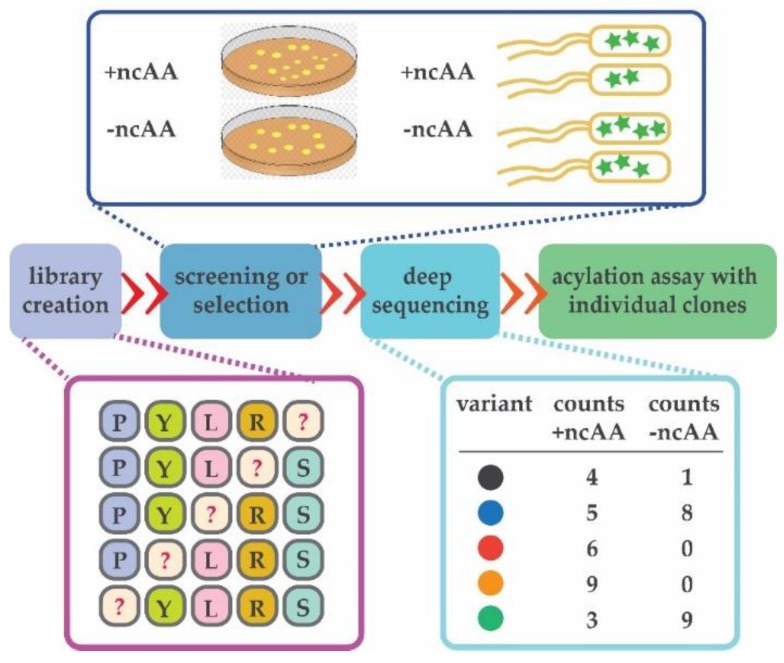
Deep mutational scanning is invaluable for the entire process of directed evolution. It can be used to inspect the quality of the initial aaRS library and quickly identify variants selective for the ncAA. Deep sequencing may be used to ensure even distribution of generated variants after mutagenesis (in purple). After screening or selection is executed in the presence and absence of an ncAA (dark blue rectangle), entire populations of cells are collected and DNA isolated. Individual variants are counted in the populations collected from both selections (light blue rectangle). Variants observed in the populations selected in the presence of ncAA can be then directly tested in an aminoacylation assay in vitro (green). Randomized positions are depicted with “?”.

Next-generation sequencing (NGS) became invaluable for directed evolution of enzymes as it allows rapid inspection of tens of thousands of protein variants [98]. In other words, the entire population of a given library can be observed before, during or after the process of directed evolution (Figure 4, [99]).

One remarkable feature of NGS-based screening in directed evolution is the ability to remove the necessity of negative selection. As mentioned earlier, reporters used in positive selection of aaRS variant mostly tolerate various AAs/ncAAs in stop codon read-through; the engineered specificity of an aaRS variant therefore depends on multiple rounds of positive and negative selections/screens in order to sift through a population containing false positives or promiscuous variants.

The possibility to remove the negative selection step may be critical for aaRS·tRNA pairs where the tRNA orthogonality is not complete, or where the starting affinity for the ncAA is very low. Orthogonality is not an absolute value; the orthogonal tRNA may be weakly recognized by some aaRS in the host, a tendency that may not be important in the presence of cognate aaRS. However, due to variation in copy numbers of plasmids from which the tRNA and aaRS are encoded, or different strength of promoters, the levels of orthogonal tRNA and aaRS may not be matched. Furthermore, if the ncAA acylation is very inefficient, the minor fraction of the orthogonal tRNA that is acylated by the host’s aaRS may become prominent. In these cases negative selection may be detrimental for orthogonal aaRS variants that have gained some improvement in ncAA acylation, but this activity is still less effective than misacylation by the host’s aaRS.

A report of special interest where NGS was employed to identify aaRS variants with improved acylation activities demonstrated that a single round of stop-codon read-through with CAT, done in parallel (with and without ncAA) allows identification of library members with improved activity towards the ncAA. Comparison between the populations growing at high chloramphenicol levels with and without the ncAA allows precise identification of the ncAA-specific library member [59].

## 4. Perspectives

Translation systems to introduce ncAAs into proteins in vivo may cause different levels of cellular toxicity. While it is possible to develop customized strains that have evolved mechanisms to help them reduce this toxicity [81], for the purposes of comprehensive aaRS engineering a more forgiving platform would be desired. In addition to the inherent lack of toxic effects, in vitro evolution of proteins allows much larger focused libraries to be tested, because the library is not limited by the transformation efficiency. Furthermore, the orthogonality requirement can be manipulated, because the canonical aaRS·tRNA pairs can be added in desired quantities and from desired sources. Finally, the ncAA concentration can be controlled far better than with in vivo systems. To date, in vitro evolution of aaRSs remains rare [100]. It is, however, foreseeable that this method might be employed more in the future. Combination of the directed evolution in vitro with ncAA-specific reporter assays in vivo may facilitate de novo protein design and the discovery of novel aaRS variants that do not resemble aaRSs found in nature.

## Abbreviations

aaRS	Aminoacyl-tRNA synthetase
AA	Amino acid
ncAA	Noncanonical amino acid
PACE	Phage-assisted continuous evolution technique
PANCE	Phage-assisted noncontinuous evolution technique
MAGE	Multiplex automated genome engineering
NGS	Next-generation sequencing
GFP	Green fluorescent protein
